# Inactive mutants of human pyridoxine 5′‐phosphate oxidase: a possible role for a noncatalytic pyridoxal 5′‐phosphate tight binding site

**DOI:** 10.1002/2211-5463.12042

**Published:** 2016-03-22

**Authors:** Mohini S. Ghatge, Sayali S. Karve, Tanya M. S. David, Mostafa H. Ahmed, Faik N. Musayev, Kendra Cunningham, Verne Schirch, Martin K. Safo

**Affiliations:** ^1^ Department of Medicinal ChemistrySchool of Pharmacy and Institute for Structural Biology, Drug Discovery and DevelopmentVirginia Commonwealth UniversityRichmondVAUSA

**Keywords:** enzyme mutation, neonatal epileptic encephalopathy, pyridoxal 5′‐phosphate, pyridoxine 5′‐phosphate oxidase, neurotransmitters, vitamin B6

## Abstract

Pyridoxal 5′‐phosphate (PLP) is a cofactor for many vitamin B6‐requiring enzymes that are important for the synthesis of neurotransmitters. Pyridoxine 5′‐phosphate oxidase (PNPO) is one of two enzymes that produce PLP. Some 16 known mutations in human PNPO (hPNPO), including R95C and R229W, lead to deficiency of PLP in the cell and have been shown to cause neonatal epileptic encephalopathy (NEE). This disorder has no effective treatment, and is often fatal unless treated with PLP. In this study, we show that R95C hPNPO exhibits a 15‐fold reduction in affinity for the FMN cofactor, a 71‐fold decrease in affinity for the substrate PNP, a 4.9‐fold decrease in specific activity, and a 343‐fold reduction in catalytic activity, compared to the wild‐type enzyme. We have reported similar findings for R229W hPNPO. This report also shows that wild‐type, R95C and R229W hPNPO bind PLP tightly at a noncatalytic site and transfer it to activate an apo‐B6 enzyme into the catalytically active holo‐form. We also show for the first time that hPNPO forms specific interactions with several B6 enzymes with dissociation constants ranging from 0.3 to 12.3 μm. Our results suggest a possible *in vivo* role for the tight binding of PLP in hPNPO, whether wild‐type or variant, by protecting the very reactive PLP, and transferring this PLP directly to activate apo‐B6 enzymes.

**Abbreviations**
NEE: neonatal epileptic encephalopathy
PLK: pyridoxal kinase
PLP: pyridoxal 5′‐phosphate
PL: pyridoxal
PMP: pyridoxamine 5′‐phosphate
PM: pyridoxamine
PNPO: pyridoxine 5′‐phosphate oxidase
PNP: pyridoxine 5′‐phosphate
PN: pyridoxine
SHMT: serine hydroxymethyltransferase

Neonatal epileptic encephalopathy (NEE) is a severe neurological disorder, which usually manifests a few hours after birth with intractable seizures that are unresponsive to conventional anticolvulsant treatment 1, 2, 3, 4, 5, 6, 7, 8, 9, 10, 11, 12, 13. Observed mainly in Turkish and Asian populations, NEE shows symptoms such as fetal distress, hypoglycemia, anemia, acidosis, and asphyxia, and most infants born with the disease die quickly, especially if left untreated 1, 2, 4, 6, 7, 10, 12, 13, 14. Analysis of cerebrospinal fluid and urine of patients shows decreased activity of vitamin B6 enzymes (PLP‐depen dent enzymes) 1, 2, 4, 6, 7, 10, 12, 13, 14. The surviving children are usually mentally retarded and most only respond to treatment with the active form of vitamin B6, pyridoxal 5′‐phosphate (PLP), but do not respond to the nutritional vitamin B6, pyridoxine 1, 2, 3, 4, 6, 7, 8, 9, 10, 11, 12, 13. This form of NEE is caused by mutations in human pyridoxine 5′‐phosphate oxidase (hPNPO) 1, 2, 3, 4, 6, 7, 8, 9, 10, 11, 12, 13, one of two enzymes that catalyze the formation of PLP *in vivo* 15, 16. The other enzyme is pyridoxal kinase (PLK) that catalyzes the phosporylation of the B6 vitamers, pyridoxal (PL), pyridoxine (PN), and pyridoxamine (PM) to PLP, pyridoxine 5′‐phosphate (PNP), and pyridoxamine 5′‐phosphate (PMP), respectively 15, 16. Both PNP and PMP require PNPO to oxidize the 4′‐alcohol or amine to form the aldehyde of PLP 15, 16. During recycling of vitamin B6 coenzymes, PLP is released from the B6 enzymes, and the 5′‐phosphate is removed by phosphatases to form PL, which is then converted back to PLP directly by PLK 15, 16. This observation explains why mutations that result in decreased or inactive forms of PNPO respond only to PLP or PL and do not respond to PN 1, 2, 3, 4, 6, 7, 8, 9, 10, 11, 12, 13.

The biosynthesis of many neurotransmitters is dependent on at least one PLP‐dependent enzyme, explaining the early manifestation of PLP deficiency such as neurological disorders. Sequencing of hPNPO coding genes in patients with NEE has identified several different pathogenic mutations, including homozygous missense (R95C, R95H, R229W, R225C, R225H), stop codon (X262Q), or splice site (IVS3‐1g > a) mutations 1, 2, 3, 4, 6, 7, 8, 9, 10, 11, 12, 13. The phenotypic effects showed by many of the mutations in PNPO are complex 7. However, the outcome of studies of dozens of individuals offer clues that mutations that most likely result in no protein being expressed were more lethal than point mutations resulting in a folded protein. What is not clear is if some of these mutations result in a partially active PNPO accounting for differences in the severity of the disease. We have previously determined the structure and catalytic activity of the R229W variant in hPNPO 9. This mutant enzyme exhibited a 862‐fold reduction in catalytic efficiency, suggesting that *in vivo* it did not make an important contribution to the level of cellular PLP 9. As expected, patients with this mutation are resistant to conventional antiseizure medication and PN, and without PLP or PL administration, the patients did not live for long 5, 7. Another point mutation that results in severe NEE, and also required administration of PLP to patients is R95C 3, 4.

In the present study, we examined the effect of the R95C mutation on the physical and catalytic properties of hPNPO. In addition, we have analyzed if the R229W and R95C mutations retain a property earlier observed with wild‐type hPNPO. Previously, both *E. coli* PNPO (ePNPO) and hPNPO have been shown to bind PLP tightly at a noncatalytic site 17, 18, 19, and ePNPO has been further shown to transfer this tightly bound PLP to an apo‐B6 enzyme, converting it to the active holo‐enzyme 19. Of importance is that in an *E. coli* extract, the purified ePNPO•PLP complex activated apo‐serine hydroxymethyltransferase (apo‐SHMT), a B6 enzyme much faster compared to the addition of an equal amount of free PLP 19. This report determines if R229W and R95C hPNPO retain this property of binding PLP tightly at a noncatalytic site; and if the wild‐type or variant hPNPO transfers the tightly bound PLP to an apo‐B6 enzyme. Finally, we also determined whether the transfer of PLP from hPNPO to B6 enzymes may involve physical interaction between the donor and acceptor enzymes.

The previous demonstration that tightly bound PLP on ePNPO transfers the PLP to an apo‐B6 enzyme *in vitro* does not confirm that this process occurs *in vivo*. The naturally occurring mutants of hPNPO that cause NEE may offer an insight into an *in vivo* role of the tightly bound PLP on hPNPO. We discuss the implications of a possible role for mutants of PNPO that retain a nearly normal structure, but have almost no catalytic activity.

## Results and Discussion

### Wild‐type and R95C hPNPO exhibit similar secondary and tertiary structures

**Figure 1:**
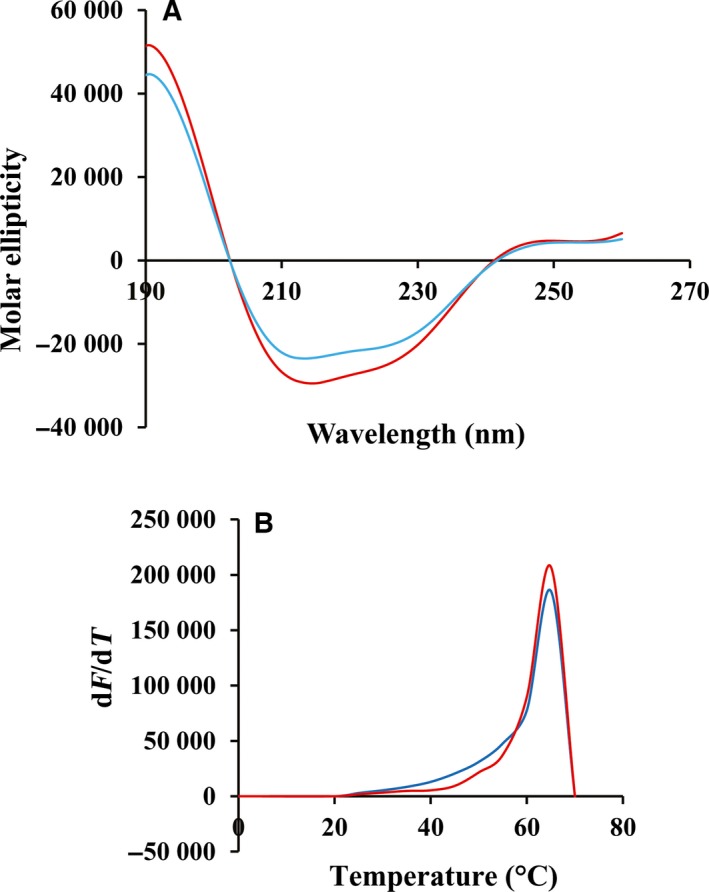
Secondary and tertiary structures of hPNPO. (A) Circular dichroism spectra of wild‐type hPNPO (cyan) and R95C hPNPO (red). (B) Melting curves of wild‐type hPNPO (cyan) and R95C hPNPO (red).

Previously, we have published the crystal structure of R229W hPNPO and showed that this mutation resulted in only minor changes in the position of a few residues at the active site of the enzyme 9. To determine if the mutation resulted in changes in the overall structure of R95C hPNPO, two experiments comparing the properties of wild‐type and R95C enzymes were performed (Fig. 1). First, circular dichroism spectra of wild‐type and R95C hPNPO exhibited identical spectra (Fig. 1A). Second, using the intrinsic fluorescence of tryptophan residues during controlled heating, each protein exhibited an unfolding temperature (*T*_m_) of 64 °C (Fig. 1B). Thus, the mutation of Arg95 to Cys does not seem to have any significant effect on the secondary and/or tertiary structure of R95C hPNPO.

### Kinetic properties of R95C hPNPO

The binding of FMN at the active site is essential for catalytic activity. Using fluorescence quenching of FMN when it binds to apo‐PNPO (no FMN bound) permits determination of the dissociation constant for this coenzyme. For wild‐type hPNPO, the dissociation constant is 24 nm, but for R95C hPNPO a value of 354 nm was obtained. This represents a 15‐fold higher dissociation constant for FMN binding to the mutant enzyme (Table 1). A similar weaker binding of FMN was reported for R229W hPNPO (a 49‐fold higher dissociation constant compared to the wild‐type enzyme) 9.

**Table 1:** Dissociation constants (*K*_d_) for FMN binding to apo‐hPNPO

Protein	*K*_d_ (nm)
Wild‐type	24 ± 3
R95C	354 ± 12

Activity measurements of wild‐type and R95C hPNPO enzymes showed a 4.9‐fold decrease in *k*_cat_ and 71‐fold increase in *K*_m_ for PNP when compared to wild‐type hPNPO, suggesting a loss of 343‐fold in catalytic efficiency as determined by *k*_cat_/*K*_m_ values (Table 2). A loss of 862‐fold in catalytic efficiency was also observed for R229W hPNPO 9. As *k*_cat_ is very low, even for the wild‐type enzyme, the value of *K*_m_ is a good estimate for the *K*_d_ value for substrate PNP. The catalytic efficiency of both R95C and R229W hPNPO may be even lower than our results suggest as in the assay we included FMN, which does not take into account the lower affinity of these mutant enzymes for FMN.

**Table 2:** Kinetic constants for hPNPO

	Wild‐type	R95C
*k*_cat_ (s^−1^)	0.18 ± 0.02	0.037 ± 0.01
*K*_m_ (μm)	6.18 ± 0.5	436 ± 35
*k*_cat_/*K*_m_ (s^−1^/μm ^−1^)	0.029	0.000085

**Figure 2:**
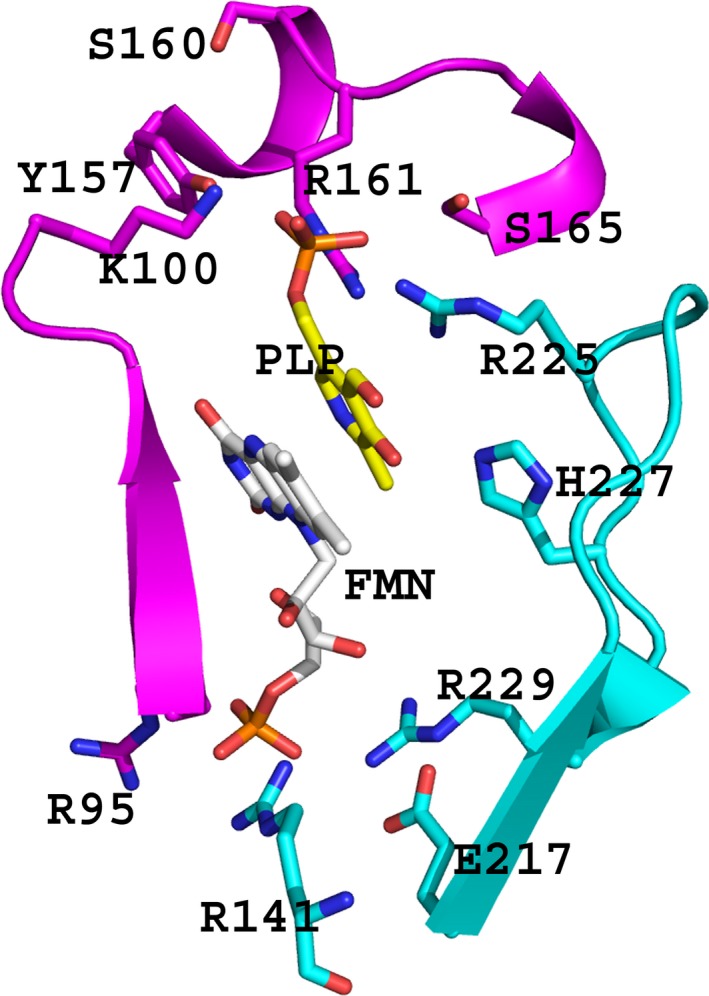
The active site of wild‐type hPNPO (PDB code 1NRG), showing bound PLP (yellow) at the *re‐*face of the FMN (gray). The two monomers, forming the homodimeric structure of the protein, are colored magenta and cyan, respectively. Note how Arg95 and Arg229 which make salt‐bridge/hydrogen interaction with FMN phosphate are far removed from the substrate binding site. For clarity, not all the residues interacting with the FMN or PLP are shown.

In the absence of the crystal structure of R95C hPNPO, the high‐resolution crystal structures of wild‐type hPNPO (1.95 Å; PDB code 1NRG) and R229W hPNPO (2.5 Å; PDB code 3HY8) 9, 17 by our group permit predictions on how the R95C mutation may explain the weaker binding of FMN. In addition to Arg95, there are three other basic residues, including Arg229, Lys117, and Arg141 that contribute to a highly conserved region of the active site of the PNPO family, all forming salt‐bridge/hydrogen bond interactions with the FMN phosphate groups to stabilize and ensure correct orientation of FMN for substrate oxidation (Fig. 2) 9, 17, 20. None of these basic residues make any direct interaction with the substrate PNP (Fig. 2). The Arg95→Cys95 exchange is expected to not only abrogate the observed bidentate salt‐bridge/hydrogen bond interactions to the FMN phosphate but also introduce a negative charge built up at the active site from the FMN phosphate; both changes likely destabilize FMN binding and explain the reduced FMN affinity.

Even though residue R229 does not make any interaction with the substrate PNP, the crystal structure of R229W hPNPO showed the mutation to have a long‐range effect on PNP binding by destabilizing the clamping effect of an active site loop on the substrate that is formed by the conserved residues Arg225 and His227 (Fig. 2) 9. Similarly, residue 95 does not make contact with the substrate (Fig. 2), but we speculate that the R95C mutation, even though located about 11 Å from the substrate binding site, may also have led to a similar substrate binding site distortion, which could decrease the enzyme binding affinity for PNP and proper orientation of the substrate, resulting in the observed catalytic deficiency.

### Tightly bound PLP in hPNPO is released slowly to solvent

Our previous studies with PNPO and PLK, 17, 18, 19, 21, and others with PLP synthase 22 have shown PLP to bind tightly to hPNPO (hPNPO*•*PLP), *E. coli* PNPO (ePNPO*•*PLP), *E. coli* PLK (ePLK*•*PLP), and plant PLP synthase (PLP synthase*•*PLP). In each case, there is one PLP bound per enzyme subunit 17, 18, 21, 22. Tight binding of PLP to these enzymes, known to bind covalently and/or not to dissociate from the enzyme during size exclusion chromatography, has been suggested as a way to prevent the highly reactive 4′‐aldehyde of PLP from spontaneously forming nondesirable or toxic adducts with non‐B6 proteins and other nucleophiles in the cell 17, 18, 19, 21, 22. This high reactivity may be the reason for the cell maintaining a low *in vivo* concentration of about 1 μm for PLP 23, 24. While PLK shows the tightly bound PLP to be at the active site, 21 its location in the oxidase is uncertain, although it has been clearly demonstrated that it is not the active site 19. The crystal structure of ePNPO revealed a second PLP bound at a site 11 Å from the active site that could be the tight binding noncatalytic PLP site 18.

**Figure 3:**
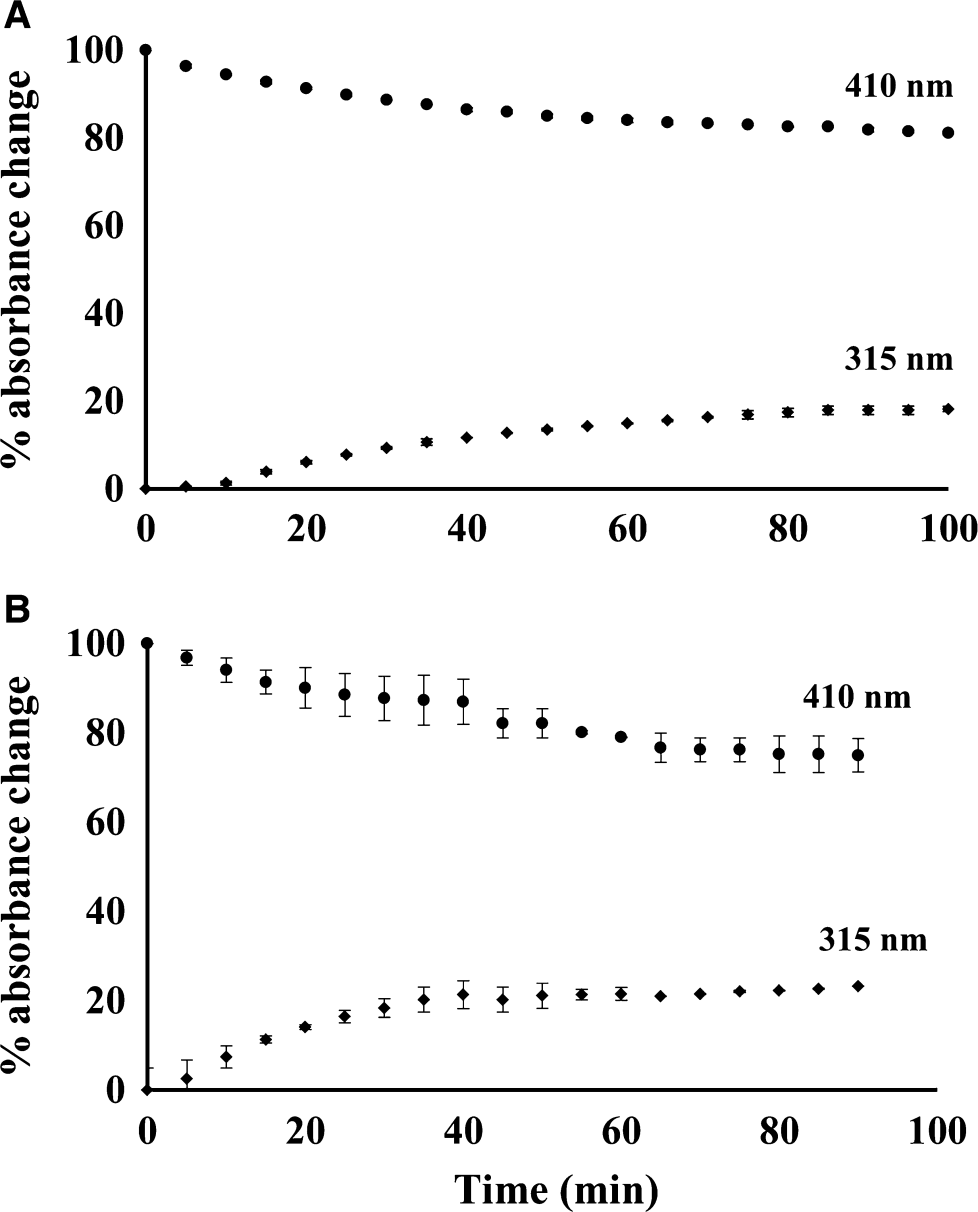
Rate of dissociation of PLP from hPNPO•PLP. Each enzyme with tightly bound PLP was incubated at 37 °C in the presence of 3 μm PLP phosphatase and the decrease in absorbance at 410 nm where protein‐bound PLP absorbs (•‐•) and increase of absorbance at 315 nm where free PL absorbs (♦‐♦) was monitored. Free PLP is rapidly converted to PL by the excess phosphatase. (A) Wild‐type hPNPO•PLP. (B) R95C hPNPO•PLP.

We have determined a value for how tightly PLP is bound in the hPNPO•PLP complex by measuring how fast PLP dissociates from hPNPO in the presence of a large excess of PLP phosphatase. The kinetics of the phosphatase reaction with free PLP shows *k*_cat_ value of 1.52 s^−1^ and *K*_m_ value of 2.5 μm 25. The phosphatase would quickly result in converting free PLP in the solvent to PL, which does not bind tightly to hPNPO. Determining the rate of loss of tightly bound PLP on wild‐type hPNPO•PLP determines the rate of dissociation of the tightly bound PLP. During a 90‐min incubation at 37^○^ only about 20% of the bound PLP was dephosphorylated to PL as indicated by the loss of absorbance at 410 nm (bound PLP) or the increase in absorbance at 315 nm (free PL) for wild‐type and R95C hPNPO (Fig. 3). We observed similar effect with the R229W hPNPO•PLP complex.

### Tightly bound PLP in the hPNPO•PLP complex is transferred to activate apo‐SHMT

**Figure 4:**
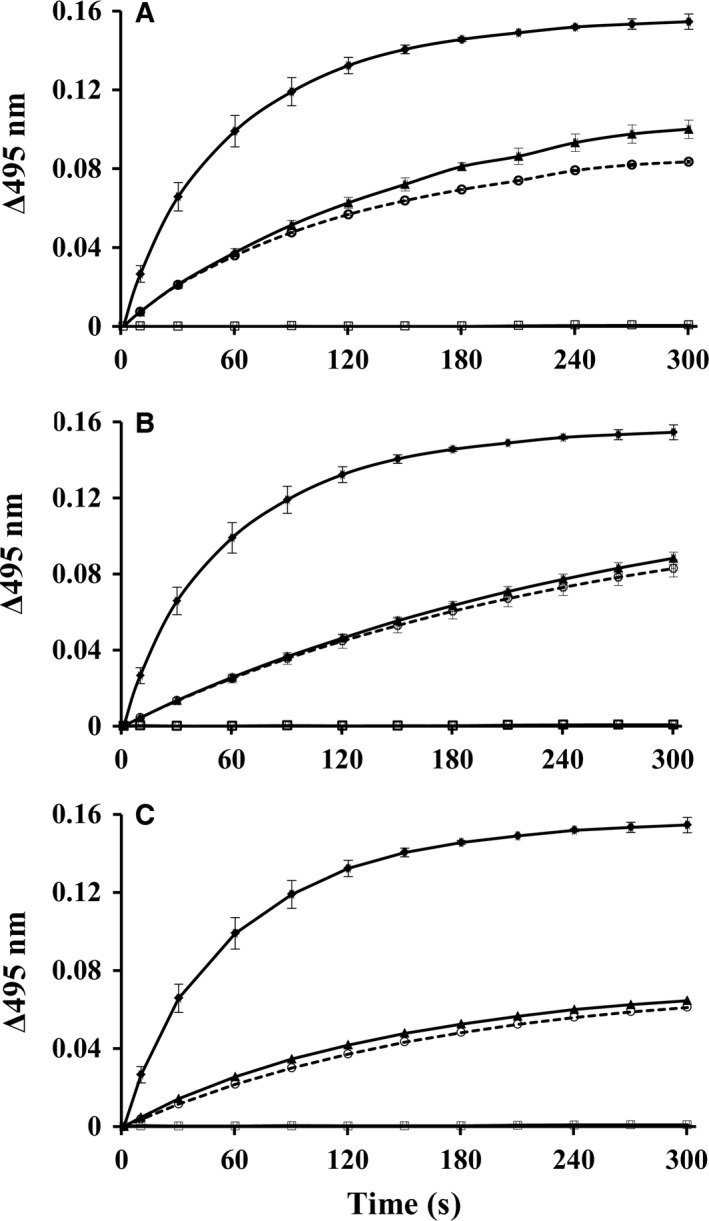
Rate of transfer of PLP from hPNPO•PLP to apo‐rcSHMT at 37 °C. In the experiment apo‐SHMT subunits and PLP are present at 20 μm concentration each. Formation of holo‐SHMT as determined by increase in absorbance at 495 nm; (♦‐♦) incubated with free PLP; (▲‐▲) incubated with hPNPO•PLP; (□‐□) incubated with free PLP in the presence of 3 μm PLP phosphatase; (○‐○) incubated with hPNPO•PLP in the presence of 3 μm PLP phosphatase. (A) Wild‐type hPNPO•PLP (B) R229W hPNPO•PLP. (C) R95C hPNPO•PLP.

We show in Fig. 4A, in a cell free system, how an equivalent amount of free PLP (20 μm) and purified wild‐type hPNPO•PLP complex (20 μm) are transferred to rabbit cytosolic apo‐SHMT (apo‐rcSHMT) (20 μm in subunit concentration). A convenient assay to determine the formation of holo‐SHMT is the rapid formation of the abortive holo‐SHMT•Gly•tetrahydrofolate complex that absorbs at 495 nm with a molar absorbance coefficient of 40 000 m ^−1^·cm^−1^ 26. This complex is formed in milliseconds once PLP is bound at the active site in a productive complex as the external aldimine. These results suggest that the rate determining step in forming holo‐SHMT is the formation of the external aldimine where PLP forms a bond with the ε‐amino group of an active Lys residue. The addition of free PLP forms the abortive complex in about 5 min (Fig. 4A ♦‐♦). When the equivalent amount of hPNPO•PLP is added to apo‐SHMT, only about 70% of the PLP from the complex is transferred to apo‐SHMT during the 5‐min incubation (Fig. 4A, ▲‐▲). The rate of forming catalytically active holo‐SHMT is slower compared to free PLP, suggesting that some aspect of the transfer is at least partially rate determining.

An unresolved question is the mechanism of transfer of PLP from the tight binding site of hPNPO to apo‐SHMT. Does the transfer occur by PLP first dissociating from the tight binding site into solvent and then binding to apo‐SHMT, or is there a direct transfer of PLP from the hPNPO•PLP tight binding site to apo‐SHMT in a complex between the two enzymes without intermediate, free PLP being involved. The rate of dissociation of tightly bound PLP in a solution containing PLP phosphatase, as shown in Fig. 3, suggests that less than 5% of the tightly bound PLP would have dissociated during the first 5 min during the transfer study. This suggests that free PLP is not an intermediate unless PLP dissociates faster from hPNPO•PLP in the presence of apo‐SHMT. However, it is possible that some contact between the two enzymes triggers the rapid release of PLP from hPNPO into solvent, which then binds to apo‐SHMT.

To test the possibility that tightly bound PLP is released rapidly, we repeated the transfer experiment by adding 3 μm PLP phosphatase to the solution of apo‐SHMT, glycine, and tetrahydrofolate before adding either free PLP or hPNPO•PLP. When free PLP was added to the assay solution, there was no formation of holo‐SHMT, showing that the phosphatase had completely converted the added free PLP to PL before it bound in a protected form on apo‐SHMT (Fig. 4A, □‐□). This suggests that the rate of binding of free PLP to the phosphatase is faster compared to the rate of binding to apo‐SHMT, where it would be protected against phosphatase action. In contrast to free PLP, when hPNPO•PLP was added to the assay solution, the phosphatase had almost no effect on the rate or extent of transfer to apo‐SHMT (Fig. 4A, ○‐○). Similar observations have been reported in studies using ePLK*•*PLP or PLP synthase*•*PLP, and the apo‐B6 enzymes, SHMT or aspartate aminotransferase 21, 22. It has also been shown that activation of apo‐SHMT in an *E. coli* extract occurs significantly faster with PNPOx•PLP compared to the addition of free PLP, which was attributed to the free PLP forming complexes with amino acids and non‐B6 enzymes 19. These observations strongly suggest that tightly bound PLP in the complexes does not dissociate into solution during transfer to apo‐B6 enzymes, implicating a likely physical contact between the donor and acceptor enzymes. Of note is that even when PLP is produced under steady‐state condition starting with PLK, PL, and ATP, apo‐B6 enzyme is still activated to the holo‐form in the presence of phosphatase, which the authors attributed to compartmentalization 27.

### Tightly bound PLP in R229W or R95C hPNPO•PLP complex is transferred to activate apo‐SHMT

For the first time, we have established that the tightly bound PLP on wild‐type hPNPO•PLP dissociates at least an order of magnitude slower compared to the rate of transfer to apo‐SHMT. We have now also established that both R95C and R229W hPNPO also bind PLP tightly. Is the tightly bound PLP on the mutant forms of hPNPO also transferred to apo‐SHMT, as observed for wild‐type hPNPO as shown in Fig. 4A? Using the same conditions as described for wild‐type hPNPO (Fig. 4A), the variants with tightly bound PLP were incubated with apo‐SHMT and the results were recorded for R95C and R229W hPNPO in Fig. 4B,C, respectively. The results are similar to those observed with the wild‐type enzyme except the rate of transfer from the mutants is slightly slower (Fig. 4B,C). Like observed with wild‐type hPNPO•PLP, only about half of the tightly bound PLP on R95C or R229W hPNPO was transferred to apo‐SHMT during the 5‐min incubation. However, the transfer of PLP for both variants was still increasing. These results confirm that both variants can bind PLP tightly at a noncatalytic site and transfer it to apo‐SHMT. The addition of phosphatase did not significantly inhibit the transfer of PLP, again suggesting that PLP is not released first to solvent before adding to apo‐SHMT.

### hPNPO forms physical complex with B6 enzymes

**Figure 5:**
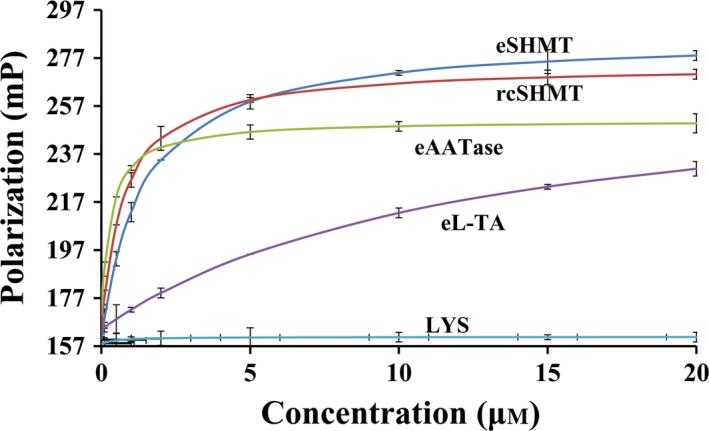
Saturation binding curves by fluorescence polarization. Titration of FMI‐tagged hPNPO with increasing concentrations of eSHMT (blue), rcSHMT (red), eAATase (green), eL‐TA (purple), and LYS (cyan).

To determine whether PNPO makes physical contact with B6 enzymes perhaps during PLP transfer, we used flourescence polarization technique to study and quantify the molecular binding interactions between wild‐type hPNPO and the following B6 enzymes, rabbit cytosolic SHMT (rcSHMT), *E. coli* SHMT (eSHMT), *E. coli* l‐threonine aldolase (eL‐TA), and *E. coli* aspartate aminotransferase (eAATase). Saturation binding curves of fluorescein 5‐maleimide (FMI)‐labeled hPNPO with the B6 enzymes clearly show interactions between the proteins as indicated by increase in polarization value with increasing concentrations of the B6 enzymes (Fig. 5). No significant change in the polarization of hPNPO was seen with lysozyme (LYS) (Fig. 5) and LDH, with similar molecular weight as the B6 enzyme (not shown), suggesting specificity of binding of hPNPO toward various B6 enzymes. A simple 1 : 1 model provided the best fit to the interactions, resulting in dissociating constant (*K*_d_) values that range from 0.3 to 12.3 μm (Table 3). Previous studies also using fluorescence polarization (FP) or surface plasmon resonance (SPR) or emission spectroscopy have reported similar complex formation between PLK and the B6 enzymes, eAATase, alanine aminotransferase, and glutamate decarboxylase, also with *K*_d_ of 2–15 μm 27, 28. These findings lend support to a direct transfer of PLP from donor to acceptor enzymes. In addition to tight PLP binding, the ability to transfer to apo‐B6 enzyme appears to be a regulatory mechanism to control the cellular content of the highly reactive PLP that otherwise if released into the solution could be destroyed by phosphatases or possibly damage the cell by binding to non‐B6 proteins or other nucleophiles.

**Table 3:** Dissociation constants (*K*_d_) for hPNPO binding to B6 enzymes

B6 enzyme	*K*_d_ (μm)
eSHMT	1.7
rcSHMT	0.8
eL‐TA	12.3
eAATase	0.3

*E. coli* SHMT (eSHMT); rabbit cytosolic SHMT (rcSHMT); *E. coli* l‐threonine aldolase (eL‐TA); *E. coli* aspartate aminotransferase (eAATase).

## Conclusion

In this study, we show that two pathogenic mutations in hPNPO (R95C and R229W), that are associated with NEE, have essentially no catalytic activity, but like the wild‐type enzyme, retain PLP tight binding and can transfer this PLP directly to apo‐SHMT without apparent release into solvent. We also show that wild‐type hPNPO forms physical complex with several B6 enzymes. Several other reported studies also show that PNPO, PLK, and PLP synthase from bacterial, animal, and plant sources all bind PLP tightly, form complexes with B6 enzymes, and are able to transfer the tightly bound PLP directly to activate apo‐B6 enzymes to holo‐B6 enzymes 17, 18, 21, 22, 27, 28, suggesting that these properties may be wide spread in nature. Nevertheless, the physiological relevance of these findings would have to be investigated with *in vivo* studies.

If direct transfer of PLP from PNPO•PLP complex to apo‐B6 enzymes is an important physiological pathway for maintaining PLP homeostasis then the finding reported here provides insight into how inactive PNPOx enzyme could still function to regulate the cellular content of PLP and its transfer to apo‐B6 enzymes. It also suggests that mutations in the recognition site between PNPO and apo‐B6 enzymes, or for binding PLP tightly in PNPO, will have physiological consequences. Further studies of the 16 mutations in hPNPO that cause NEE will help determine if their pathogenic effects are the result in loss of catalytic activity of hPNPO or possibly either the loss of the ability to bind PLP tightly or to transfer it to apo‐B6 enzymes. As NEE becomes an increasingly recognized cause of seizures in newborn children, and PLP therapy becomes more standardized, the effectiveness as a therapy for the different mutations may result in different treatment protocols. As one example, our studies suggest that for the R95C and R229W mutations, addition of riboflavin in the diet may have a modest beneficial effect as both mutations significantly decrease the affinity of hPNPO for FMN.

## Experimental procedures

### Materials

All the chemicals, reagents, buffers, etc. used in this study were of best purity available from Fisher Scientific (Hampton, NH, USA), Sigma‐Aldrich (St. Louis, MO, USA), and Qiagen (Venlo, the Netherlands). PLP phosphatase was expressed and purified as described previously 21.

### Preparation of wild‐type, R229W, and R95C hPNPO

Wild‐type and R229W hPNPO were expressed and purified following previously described procedures 9, 17. The R95C hPNPO was made using the cDNA coding for the wild‐type hPNPO. A standard protocol, described in the QuickChange kit from Stratagene (Agilent, Santa Clara, CA, USA) was followed up for the site‐directed mutagenesis using primers: Forward: 5′‐ GGA AAA CCC TCT GCT **TGC** ATG TTG CTG CTG AAG ‐ 3′ and reverse: 3′‐ CCT TTT GGG AGA CGA **ACG** TAC AAC GAC GAC TTC ‐ 5′. The underlined nucleotides correspond to codon changes for the Arg to Cys mutation. The PCR product was transformed into chemically competent *E. coli* Rosetta (λDE3) pLysS cells. Transformants were grown at 37 °C in LB medium till OD_600 nm_ reached 1.0, followed by induction with 50 μm IPTG overnight at 18 °C. Cells were harvested by centrifugation and then resuspended in cold 50 mm potassium phosphate buffer at pH 7.4, containing 300 mm NaCl, 5 mm 2‐mercaptoethanol, and 10 μm FMN. Cell lysis was carried out using a French press. The cell debris was separated by centrifugation. PNPO was then purified as previously described 9, 17. Protein concentrations were determined using the molar extinction coefficient of 76 760 cm^−1^·m ^−1^ at 280 nm.

### PNPO activity assay

The activities of wild‐type, R95C, R229W hPNPO enzymes were followed using PNP as the substrate as described previously 9, 17. The assay was performed in a 10‐cm pathlength cuvette at 37 °C, in 50 mm Tris–HCl buffer at pH 8, containing 10 μm FMN and 1 mm dithiothreitol (DTT). Product PLP rapidly forms an aldimine with the amino group of Tris (as the buffer) that absorbs at 414 nm with a molar absorbance coefficient of 5900 cm^−1^·m ^−1^. Substrate PNP concentrations varied between 0.01 and 2 mm. Initial velocities were determined for the first 5% of the reaction. *K*_m_ and *k*_cat_ values were determined from double reciprocal plots of the initial velocities and substrate PNP concentrations.

### Determination of dissociation constant (*K*_d_) of FMN binding to R95C apo‐hPNPO

Apo‐hPNPO was prepared by removing the bound FMN as previously described 9, 29. Quenching of FMN fluorescence upon binding to apo‐PNPO was followed to determine the dissociation constant (*K*_d_) for FMN binding to R95C hPNPO as previously described for the wild‐type and R229W hPNPO 9.

Briefly, varying concentrations of R95C apo‐PNPO enzyme was added to 50 nm FMN in 50 mm potassium phosphate buffer (pH 7.2) containing 1 mm DTT. Excitation wavelength was set at 450 nm with excitation slit of 1 nm on a Shimadzu RF‐5301 PC. Fluorescence emission spectra for FMN between 470 nm and 570 nm, with a 10‐nm emission slit, were recorded using a 1‐cm pathlength quartz cell. *K*_d_ value was calculated based on equation (1) 30, where Δ*F/F*_o_ is the fractional fluorescence change at 525 nm at varying concentrations of the apo‐enzymes, *F*_O_ is the fluorescence in the absence of apo‐enzyme, Δ*F*_max_ is maximum change in fluorescence intensity, [APO] is the total apo‐enzyme concentration, [FMN] is the total cofactor concentration, *K*_d_ is the dissociation constant of the equilibrium:

APO+FMN⟷HOLO,

(1) $$\frac{\Delta F}{F_{o}}=\Delta F_{\text{max}}\;\;*\;\;\frac{\sqrt{{\left(\left[\text{APO}\right]+\left[\text{FMN}\right]+K_{d}\right)}^{2}-\left(4*\left[\text{FMN}\right]*\left[\text{APO}\right]\right)}}{2*[\text{FMN}]}.$$

### Secondary structure analysis of PNPO

Far‐UV cirular dichroism (CD) spectra were determined using an Olis optical spectropolarimeter with a 0.5‐cm pathlength cuvette. Wavelength scans were obtained from 190 to 260 nm for both wild‐type and R95C hPNPO diluted in 50 mm sodium phosphate buffer, pH 7.3 to a final concentration of 0.1 mg·mL^−1^.

### Determination of hPNPO stability

Protein solutions of wild‐type and R95C hPNPO enzymes (3.5 μm) in 50 mm sodium HEPES buffer pH 7.2, containing 0.2 μm DTT and 0.1 μm EDTA in 0.5‐mL cuvette, were heated from 20 to 70 °C at the rate of 5 °C per minute using a circulating water bath. Fluorescence spectra of samples at all temperatures were recorded on a fluorescence spectrophotometer by Photon Technology International. The excitation wavelength was set at 280 nm. Emission spectra were recorded from 300 to 380 nm with excitation slit of 2.5 nm and emission slit of 5 nm. The change in tryptophan fluorescence at 329 nm was noted and a melting curve was plotted. A derivative plot of the melting curve was then used to determine the melting temperature (*T*_m_) of both proteins.

### Preparation of hPNPO•PLP complexes

The complex hPNPO•PLP, either from the wild‐type, R229W, or R95C hPNPO enzyme, was prepared as described previously by incubating 150 μm of the protein with a three‐fold molar excess of PLP in a 500‐μL reaction in 20 mm potassium phosphate buffer, pH 7.3 with 5 mm 2‐mercaptoethanol at room temperature for 30 min 19. The sample was loaded onto a 0.8 × 29 cm column of Biogel P6 equilibrated with 20 mm potassium phosphate buffer, pH 7.3 containing 5 mm 2‐mercaptoethanol. Equilibration buffer was passed through the column to separate free PLP from the protein PLP complex. Aliquots of 0.8‐mL fractions were collected and monitored for protein at 280 nm and PLP at 388 nm. The stoichiometry of PLP binding was determined as described previously 19.

### Preparation of rcSHMT and apo‐rcSHMT

Rabbit cytosolic serine hydroxymethyltransferase was expressed and purified following a previously described procedure 31. Apo‐rcSHMT was prepared with modifications 32. Briefly, 10 mg·mL^−1^ pure rcSHMT was incubated in 50 mm potassium phosphate buffer pH 7.6 containing 1 mm DTT with 200 mm d‐alanine and 200 mm ammonium sulfate at 37 °C for 2–3 h, upon which the yellowish rcSHMT changed to the colorless apo‐rcSHMT as a result of the half‐transamination of d‐alanine to pyruvate and PLP to pyridoxamine 5′‐phosphate (PMP). The apo‐enzyme preparation was then dialyzed extensively against 50 mm potassium phosphate buffer pH 7.3 containing 0.1 mm EDTA and 5 mm β‐mercaptoethanol. Fresh apo‐rcSHMT preparation was used for transfer studies and any remaining preparation was stored at −80 °C with 5% sucrose.

### Rate of dissociation of tightly bound PLP from hPNPO•PLP complex

The rate of dissociation of PLP from hPNPO*•*PLP either from the wild‐type or R95C/R229W hPNPO was determined by the rate of loss of absorbance at 410 nm (bound PLP) and the increase in absorbance at 315 nm (free PL). For each study, the enzyme–PLP complex (20 μm) in 50 mm potassium phosphate, pH 7.3, buffer was added to a cuvette followed by 3 μm PLP phosphatase (in the same buffer) and decrease in the absorbance at 410 nm was monitored every 5 min for 90 min at 37 °C. When PLP is released from the tight binding site on hPNPO, it will be rapidly dephosphorylated by the phosphatase to PL.

### Transfer of the tightly bound PLP from hPNPO•PLP to activate apo‐SHMT

The hPNPO•PLP complex, either from wild‐type or R95C/R229W hPNPO, was used as a source of PLP to activate apo‐rcSHMT to holo‐rcSHMT. In a 1‐mL cuvette containing 50 mm BES, pH 7.5, 5 mm 2‐mercaptoethanol, 50 mm glycine, 100 μm tetrahydrofolate, and 20 μm apo‐SHMT at 37 °C, 20 μm of hPNPO*•*PLP was added, and the transfer of PLP from the hPNPO•PLP complex was then followed by measuring the formation of the abortive holo‐SHMT•Gly•tetrahdyrofolate complex that absorbs at 495 nm with a molar absorbance value of 40 000 m ^−1^·cm^−1^ 26. This complex is formed in milliseconds with holo‐SHMT. To determine the fraction of holo‐SHMT formed, a control experiment was performed where the hPNPO*•*PLP complex was replaced with 20 μm free PLP, which would fully saturate the apo‐SHMT. The above experiments were duplicated in the presence of 3 μm PLP phosphatase that was added to the reaction solution prior to starting the transfer with either free PLP or hPNPO*•*PLP.

### Labeling of wild‐type hPNPO for fluorescence polarization experiment

Human PNPO (50 μm) was mixed with 1 mm fluorescein 5‐maleimide (FMI) (Invitrogen, Part of Thermo Fisher, Waltham, MA, USA) in 1 mL of 50 mm sodium HEPES buffer, pH 7.55 containing 150 mm KCl and 0.01% Triton. The reaction was allowed to occur overnight at 4 °C in the dark, then centrifuged and the supernatant dialyzed against the same buffer in dark overnight to remove the excess dye. The FMI‐labeled protein was stored at −20 °C in amber colored container when not in use. The degree of labeling of hPNPO was determined using the molar extinction coefficient of 68 000 m ^−1^·cm^−1^ at 495 nm. The activity of hPNPO was not affected by the labeling.

### Determination of *K*_d_ of hPNPO binding to B6 enzymes

Fluorescence polarization technique was used to quantify the interactions between hPNPO and the B6 enzymes, eL‐TA, eAATase, eSHMT, and rcSHMT. As a negative control, binding curves of non‐vitamin B6 enzymes, lactate dehydrogenase (LDH), and lysozyme with hPNPO were also obtained. The assay was performed using Tecan Polarion polarimeter. The excitation wavelength was set at 495 nm and emission wavelength at 535 nm. FMI‐labeled hPNPO (1 μm) was added to separate wells in a 96‐well round bottom polystyrene opaque plate Model # 3792 by Corning Inc (Corning, NY, USA). The B6 enzymes were added to the wells at concentrations ranging from 0 to 20 μm, and the plate incubated at 25 °C for 5 h. Following, the FP of the protein mixtures was determined and the polarization values (in mP) were directly read from microsoft excel spreadsheet interfaced with the polarimeter. Labeled hPNPO was used as a blank. The mP values were plotted against concentrations of B6 enzymes. The dissociation constants for the protein–protein complexes were obtained by fitting the curves to equation (2) using sigmaplot 11 (SYSTAT, San Jose, CA, USA);

(2) $$P_{\text{obs}}=\frac{\left(P_{0}*K_{d}+2P_{\max}*\left[E\right]\right)}{\left(K_{d}+2\left[E\right]\right)}.$$

where, *P*_obs_ is the observed polarization value, *P*_0_ is the polarization value when no B6 enzyme was added, *P*_max_ is the maximum polarization value obtained upon saturation of the polarization curve, *K*_d_ is the dissociation constant for the protein–protein complex and *[E]* is the concentration of B6 enzymes. The above equation was derived from the equation (3) 33 by assuming the equilibrium:

FMI‐PNPO+E⟷FMI‐PNPO‐E

(3) $$P_{\mathrm{obs}}=\frac{\left(\left[\text{FMI-PNPO}\right]*P_{\mathrm{FMI}-\mathrm{PNPO}}+\left[\text{FMI-PNPO-E}\right]*P_{\mathrm{FMI}-\mathrm{PNPO}-E}\right)}{\left(\left[\text{FMI-PNPO}\right]+\left[\text{FMI-PNPO-E}\right]\right)}$$

where, *P*_obs_ is the observed polarization values, *P*_FMI‐PNPO_ is polarization intensity of labeled hPNPO, *P*_FMI‐PNPO‐E_ is the polarization values of the complex of labeled hPNPO and B6 enzyme, when all the hPNPO is in the complexed form. [FMI‐PNPO] and [FMI‐PNPO‐E] are the concentrations of noncomplexed and complexed labeled hPNPO, respectively.

## Author contributions

MSG performed molecular biology, kinetic experiments, and biophysical experiments; SSK performed molecular biology, kinetic, and biophysical experiments; TMSD performed molecular biology experiments; MHA performed biophysical experiments; FNM performed molecular biology experiments; KC performed molecular biology and kinetic experiments; VS performed kinetic experiments and wrote paper; and MKS coordinated the project and wrote paper.
